# Accessibility of ophthalmic healthcare for residents of an offshore island—an example of integrated delivery system

**DOI:** 10.1186/s12913-016-1501-8

**Published:** 2016-07-13

**Authors:** Li-Ju Chen, Yun-Jau Chang, Chun-Fu Shieh, Jy-Haw Yu, Ming-Chin Yang

**Affiliations:** Institute of Health Policy and Management, College of Public Health, National Taiwan University, Room 637, No 17, Hsu-Chow Road, Taipei, 10055 Taiwan; Department of Ophthalmology, Heping Branch, Taipei City Hospital, Taipei, Taiwan; Department of General Surgery, National Taiwan University Hospital, Taipei, Taiwan; Department of General Surgery, Zhongxing branch, Taipei City Hospital, Taipei, Taiwan; Public Health Bureau, Lienchiang County, Matsu Taiwan

## Abstract

**Background:**

To assess the utilization of and satisfaction with ophthalmic healthcare provided by integrated delivery system (IDS) since 2000 and vision-related quality of life (VRQoL) for residents of an offshore island of Taiwan.

**Methods:**

Facilitators interviewed residents (age ≥ 50 years) with the 25-item National Eye Institute Visual Function Questionnaire (NEI-VFQ-25) for VRQoL and a questionnaire on clinical information, ophthalmic care utilization and satisfaction.

**Results:**

A total of 841 participants (response rate 93.4 %, 841/900) completed the questionnaire survey. Mean age was 63.7 (±10. 7) years. The common eye diseases were cataract (44.7 %), dry eye (15.5 %), and glaucoma (8.7 %). Among the participants, 61.0 % sought ophthalmic care under the IDS in the past year and 17.6 % experienced unmet ophthalmic needs in the past 6 months. Satisfaction with ophthalmic care under the IDS was 88.1 %. Determinants of dissatisfaction under the IDS were distance to healthcare facility and VRQoL. Predictors of VRQoL included age, residential area, marital status, occupation, comorbid condition, commercial insurance, household income, cataracts and glaucoma.

**Conclusions:**

The implementation of IDS improves accessibility of ophthalmic care for residents of an offshore island. Geographic proximity to avail healthcare facility and VRQoL affect satisfaction with the IDS.

## Background

An integrated delivery system (IDS) is a collaborative network of organizations that provides or arranges to provide a coordinated continuum of services to a defined population and is willing to be held clinically and fiscally accountable for the outcomes and health status of the population served [1]. In areas where physicians are hard to find, the introduction of IDS may be one of the best solutions to overcome the dilemma of specialist shortages and geographic barriers. Studies have shown that residents of remote areas had lower healthcare utilization compared to residents of urban areas and that this difference could probably be attributed to inequality of access [2–4]. Access to healthcare is important and measurable in the performance of health systems around the world. Andersen’s behavior model provides measures of access to medical care [5]. The framework divides access into two dimensions: potential and realized access [6]. Potential access is defined as the system availability, community characteristics, individual predisposing characteristics (such as age, sex, race, education, occupation and health habits), individual enabling characteristics (such as income, insurance, and travel time), and individual need (such as perceived health, worry and symptoms) [6, 7]. Realized access is the actual use of services and is measured by an objective indicator (utilization) and a subjective indicator (consumer satisfaction) [7]. Although the objective of IDS in healthcare services is explicit, the outcomes of accessibility after its implementation in medically underserved areas such as an offshore island have not yet been well established through empirical research.

The National Health Insurance (NHI) program in Taiwan (instituted in 1995) is a single-payer system that covers 99 % of the population with an average of 80 % satisfaction [8–10]. Coverage has reached 100 % since 1999 for the aborigines and residents of offshore islands after the government subsidized their health insurance premium [11]. The residents living in rural districts are also exempted from copayment when they seek healthcare at their local facilities [11]. This NHI program has attracted worldwide attention due to its success in both stabilizing healthcare expenses and reducing health disparity across various socioeconomic gradients [12–15]. However, the distribution of physicians and of medical equipments tends to be centralized. Taiwan consists of a number of sparsely populated mountainous areas and islands where natural factors limit the willingness of healthcare providers to work there and therefore the residents of such regions suffer from interrupted healthcare services. The accessibility of medical facility is thus a main concern of people in rural areas.

To overcome the geographic barrier and offer easy access to medical services, the Bureau of NHI initiated an IDS program in November 1999 that currently covers all mountainous and outlying islands in the country to provide a continuum of healthcare services [16]. Matsu Archipelago, a former military stronghold and historic area of Taiwan, lies almost 200 km from Taiwan proper across the Taiwan Strait. The transportation of residents and travelers depends solely on planes or ferries, and unfortunately, it is often hampered by foggy or bad weather. Therefore, general practitioners in Matsu are relatively scarce, let alone specialists such as ophthalmologists. To improve accessibility, Taipei City Hospital contracted the IDS program and began rotating its ophthalmologists to Matsu in 2000 to provide ophthalmic services (6 days per fortnight), excluding intraocular and laser surgery [8]. During the week without an ophthalmologist, eye services were delivered by local general practitioners. In addition, the Health Promotion Administration has launched a community-based integrated screening project that has included annual eye screening for cataracts and glaucoma in Matsu since 2002. Nevertheless, residents’ vision-related quality of life (VRQoL) and ophthalmic accessibility after IDS implementation in Matsu have never been investigated.

This study aimed to assess the ophthalmic accessibility of residents in an outlying island under the provision of IDS. Utilization and satisfaction measures were used as indicators of actual, or realized, access [7]. We also investigated VRQoL via the 25-item National Eye Institute Visual Function Questionnaire (NEI-VFQ-25) and explored the associations between frequently seen eye diseases and VRQoL on a community basis.

## Methods

Residents of Nangan (major island of Matsu) who were 50 years of age or older were identified as eligible to participate this study, which was conducted from May to June 2014. They accounted for more than 60 % of the population of Matsu, according to the national census [17]. The age selection criterion was based on evidence that two-thirds of the visual impairment (including blindness) worldwide occurs in this age group [18]. The participants were required to have sufficient cognitive ability to understand and complete the face-to-face survey. Experienced interviewers were selected from local residents and attended seminars hosted by one of the authors beforehand to facilitate the survey. The questionnaire consisted of two parts: the first part was NEI-VFQ-25, and the second was a constructed questionnaire about socioeconomic status, clinical information, the utilization of and satisfaction with ophthalmic healthcare. All participants completed these questionnaires anonymously. The institutional review board of Taipei City Hospital approved this survey and waived the signed consent sheet (TCHIRB-1020122-E). The study was conducted in accordance with the tenets of the Declaration of Helsinki.

The NEI-VFQ-25 is the most commonly used patient-reported outcome measure to assess vision-targeted functioning and well-being based on factors identified as important by persons with various chronic eye diseases [19–21]. It consists of 25 core items to measure 12 domains of visual function. The NEI-VFQ-25 Taiwan Chinese version has been formatted according to standard procedures (including forward translation, backward translation, examination of the translation quality by bilingual speakers, and a pilot test) [22]. The NEI-VFQ-25 scores were analyzed and converted to a 100-point score in which 100 represented the best possible and 0 represented the worst. Twelve subscale scores and a single composite score were calculated according to the standard algorithm for scoring [21]. The composite score was derived from the average of subscale scores excluding “general health” [23, 24] and “driving” [19, 22] because more than 40 % of participants did not drive. Likewise, the composite score ranged from 0 to 100, with higher scores indicating better quality of life. The other part of the questionnaire originated from the National Health Interview Survey provided by the National Health Research Institutes [25]. Information regarding socio-demographic data (age, gender, residential village, religion, education level, occupation, marital status, annual household income), unmet needs to seek ophthalmic care and the utilization of and satisfaction with ophthalmic care were also included. The questionnaires were scrutinized by 5 experts for content validity.

Dependent variables included the utilization of ophthalmic care, satisfaction with ophthalmic care and VRQoL (composite score of NEI-VFQ-25). An unmet ophthalmic need was defined as residents not consulting eye doctors despite eye discomfort (such as blurred vision, aching, itching or burning sensation) within the past half year. An ophthalmic referral was defined as a referral (to Taiwan proper) made by ophthalmologists or physicians for participants due to ophthalmic problems. Predisposing (age, gender, religion, education, marital status, occupation, duration of residence, health habits) and enabling factors (residential village, annual household income, commercial insurance) were considered as predictors. Continuous variables were compared using Student’s *t* test. Categorical variables were analyzed using the *X*^2^ test. The logistic regression method was used when the dependent variable was dichotomous. Linear regression was used when the dependent variable was continuous. A *P* value below 0.05 (two-tailed) was considered statistically significant. The reliability of the questionnaires was assessed using Cronbach’s α test.

## Results

Nine hundred questionnaires were administered during the study period and a total of 851 questionnaires were returned. Ten questionnaires did not contain enough information for this study and were subsequently excluded. In the end, 841 questionnaires were considered valid and eligible for this study (response rate 93.4 %). These participants accounted for 63 % of the entire eligible population. The socio-demographic characteristics of these participants are summarized in Table 1. The participant age ranged from 50 to 101 years with a mean age of 63.7 (±10.7) years. Females comprised 51.5 % of the participants and predominated in each age group. Most (84.3 %) participants had lived on Matsu Island for more than 20 years, and 49.4 % of the participants lived in the neighborhood of the healthcare facility. The education level of 32.7 % was at least senior high school, while 23.3 % of the participants were illiterate. A total of 45.1 % of participants had private medical insurance. The annual household income was lower than 500,000 New Taiwan dollars for more than half of participants.

**Table 1:** Socio-demographic characteristics, health habits, and illness characteristics of study participants (*N* = 841)

Variable	Number	Percent
Sex		
Male	408	48.5
Female	433	51.5
Age (years)		
63.7 (50–101)	±10.7	–
Residential village		
Neighborhood of healthcare facility	415	49.4
Other area	426	50.6
Years of residence in Matsu		
< 20 years	132	15.7
20–40 years	71	8.4
> 40 years	634	75.4
Unspecified	4	0.5
Religion		
No	55	6.5
Traditional folk belief	621	73.8
Buddism/Taoism	138	16.4
Christianity	18	2.1
Others	9	1.1
Education		
Primary school completed or less	459	54.6
Junior high school	105	12.5
Senior high school	215	25.6
College or higher	60	7.1
Unspecified	2	0.2
Marital status		
Never married	15	1.8
Married or cohabiting	663	78.8
Divorced or separated	26	3.1
Widowed	113	13.4
Unspecified	24	2.9
Main occupation		
Housekeeper	146	17.3
Public servant	126	15.0
Teacher	8	1.0
Worker, farmer or fisher	185	22.0
Businessman	76	9.0
Other	77	9.2
No/unemployment/retired	218	25.9
Unspecified	5	0.6
Commercial insurance		
Yes	379	45.1
No	462	54.9
Annual household income		
≤ NTD 500,000	434	51.6
> NTD 500,000, ≤ 800,000	165	19.6
> NTD 800,000, ≤ 1,000,000	118	14.0
> NTD 1,000,000, ≤ 1,500,000	85	10.1
> NTD 1,500,000	33	3.9
Unspecified	6	0.7
Alcohol consumption		
No	478	56.8
Rare (<1 time/month)	97	11.5
Yes	266	31.6
Tobacco use		
Never	696	82.8
Quit	41	4.9
Current	104	12.4
Daily sun exposure time (hours)		
< 3	624	75.1
3 ~ 5	109	13.1
> 5	98	11.8
Comorbid condition (multiple)^a^		
None	304	36.1
Hypertension	439	52.2
Rheumatoid arthritis	163	19.4
Diabetes	99	11.8
Heart disease	72	8.6
Asthma or chronic lung disease	25	3.0
Others	50	5.9
Eye diseases diagnosed by ophthalmologist (multiple)^a^		
None	311	37.0
Cataract	376	44.7
Cataract (mild)	259	30.8
Cataract (moderate or severe)	117	13.9
Dry eye	130	15.5
Glaucoma	73	8.7
Retinal disease	41	4.9
High myopia (≤ − 6 Diopters)	34	4.0
Others	58	6.9
Total	841	100

*Abbreviations*: *NTD* New Taiwan dollar

^a^indicate numbers would sum up greater than 841

Common comorbid conditions included hypertension (52.2 %), arthritis (19.4 %) and diabetes mellitus (11.8 %). Common eye diseases included cataract (44.7 %), dry eye (15.5 %) and glaucoma (8.7 %). Sixty-one percent of participants had utilized the IDS in the past year (Table 2). Only 14 % of participants had a history of being referred to Taiwan, traveling by planes or ferries, for further eye care, and 18 % of participants had unmet ophthalmic care needs during the past 6 months. Most participants (94 %) considered eye healthcare in Matsu to be accessible and convenient, as 87 % of participants spent twenty minutes or less to reach eye care. Overall, 88 % of the participants were satisfied with the eye care provided by the IDS.

**Table 2:** Utilization and satisfaction with ophthalmic care of study participants (*N* =841)

Variable	Number	Percent
Ophthalmic utilization		
Visit IDS ophthalmic clinic in the past year		
Yes No	513 328	61.0 39.0
History of ophthalmic referral		
Yes No	115 726	13.7 86.3
Unmet ophthalmic need within the past six months^b^		
Yes No	148 693	17.6 82.4
Ophthalmic satisfaction		
Satisfaction^a^		
Satisfied Dissatisfied	741 100	88.1 11.9
Total	841	100

*Abbreviations*: *IDS* Integrated delivery system

^a^Opinion about ophthalmic care services by the participants who used ophthalmic care services

^b^An unmet ophthalmic need was defined as residents not consulting eye doctors despite eye discomfort (such as blurred vision, aching, itching or burning sensation) within the past 6 months

Predisposing factors (age, duration of residence, occupation) and comorbid conditions were associated with ophthalmic referrals. Older age, longer years of residence, the non-working group, and more (≥ 2) comorbid conditions were associated with more ophthalmic referrals compared to their counterparts (odds ratio (OR) 3.23, 95 % confidence interval (CI) 2.04–5.00, *P* < 0.001; OR 2.40, 95 % CI 1.36–4.23, *P* < 0.001; OR 2.38, 95 % CI 1.59–3.57, *P* < 0.001; OR 2.82, 95 % CI 1.88–4.22, *P* < 0.001, respectively; see Table 3, Model A). Predisposing (age, duration of residence, marital status, religion) and enabling (residential village) factors were associated with the utilization of the IDS ophthalmic clinic in the past year. Older age, living in the neighborhood of the healthcare facility, longer years of residence, being married or cohabiting, and being Buddhist/Taoist were associated with more utilization of ophthalmic visits than their counterparts (OR 1.39, 95 % CI 1.05–1.82, *P* = 0.020; OR 2.13, 95 % CI 1.64–2.86, *P* < 0.001; OR 1.79, 95 % CI 1.30–2.45, *P* < 0.001; OR 1.56, 95 % CI 1.14–2.17, *P* = 0.008; and OR 1.72, 95 % CI 1.03–2.78, *P* = 0.019, respectively; see Table 3, Model B). Predisposing factors (age, occupation), comorbid conditions, and enabling factor (commercial insurance status) were associated with unmet ophthalmic needs. Older age, the non-working group, no commercial insurance and more (≥2) comorbid conditions were associated with more unmet ophthalmic needs compared to their counterparts (OR 1.45, 95 % CI 1.01–2.08, *P* = 0.04; OR 1.49, 95 % CI 1.04–2.13, *P* = 0.027; OR 1.79, 95 % CI 1.22–2.56, *P* = 0.002; OR 1.61, 95 % CI 1.10–2.33, *P* = 0.014, respectively; see Table 3, Model C).

**Table 3:** Multiple logistic regression of association between predisposing and enabling factors and utilization (model A: referral; model B: seek eye care within the past year; model C: unmet ophthalmic need)

	Model A: History of ophthalmic referral			Model B: Visit IDS ophthalmic clinic within the past year			Model C: ^a^Unmet ophthalmic need		
Predictors	OR	95 % C.I.	*P* value	OR	95 % C.I.	*P* value	OR	95 % C.I.	*P* value
Sex (male)	1.03	(0.70–1.54)	0.867	0.93	(0.49–1.11)	0.599	1.02	(0.71–1.45)	0.915
Age (50–60 yrs)	3.23	(2.04–5.00)	< 0.001	1.39	(1.05–1.82)	0.020	1.45	(1.01–2.08)	0.040
Village (not neighborhood of healthcare facility)	0.97	(0.65–1.45)	0.871	2.13	( 1.64–2.86)	< 0.001	1.10	(0.77–1.56)	0.611
Years of residence (< 40)	2.40	(1.36–4.23)	< 0.001	1.79	( 1.30–2.45)	< 0.001	1.49	(0.96–2.33)	0.082
Education (≤ junior high school)	0.88	(0.58–1.35)	0.514	0.76	( 0.57–1.02)	0.066	0.79	(0.53–1.15)	0.182
Marital status (not married/cohabiting)	0.90	(0.56–1.45)	0.677	1.56	( 1.14–2.17)	0.008	0.97	(0.63–1.49)	0.898
Occupation (working group)	2.38	(1.59–3.57)	< 0.001	1.16	( 0.88–1.54)	0.277	1.49	(1.04–2.13)	0.027
Religion (non-Buddist or Taoist)	0.74	(0.40–1.35)	0.320	1.72	( 1.03–2.78)	0.019	1.25	(0.66–2.38)	0.489
Annual household income (≤ NTD 500,000)	1.28	(0.86–1.89)	0.215	0.97	( 0.74–1.28)	0.792	0.77	(0.58–1.19)	0.313
Commercial insurance (yes)	1.41	(0.94–2.13)	0.094	0.83	( 0.63–1.09)	0.179	1.79	(1.22–2.56)	0.002
Daily sun exposure time (< 3 hours)	1.08	(0.68–1.69)	0.747	1.15	( 0.84–1.59)	0.371	1.05	(0.70–1.59)	0.805
Comorbid condition (< 2 diseases)	2.82	(1.88–4.22)	< 0.001	1.20	( 0.88–1.64)	0.247	1.61	(1.10–2.33)	0.014
Alcohol (no)	0.72	(0.48–1.09)	0.122	0.97	( 0.74–1.28)	0.861	1.22	(0.85–1.75)	0.263
Tobacco (no)	0.76	(0.49–1.33)	0.342	0.61	( 0.42–0.87)	0.007	1.05	(0.66–1.67)	0.841

Items in parentheses indicate reference group

*Abbreviations*: *IDS* Integrated delivery system, *NTD* New Taiwan dollar

^a^An unmet ophthalmic need was defined as residents not consulting eye doctors despite eye discomfort (such as blurred vision, aching, itching or burning sensation) within the past 6 months

Table 4 summarizes the results of the univariate and multivariate logistic regressions on dissatisfaction. The significant predictors of dissatisfaction were the neighborhood of the healthcare facility and the composite NEI-VFQ-25 score. Participants who did not live in the neighborhood of the facility were more dissatisfied than participants who lived in the neighborhood (OR 3.88, 95 % CI 2.85–5.28, *P* < 0.001). Participants with a higher composite NEI-VFQ-25 score (> 85) were less dissatisfied than participants with a lower composite score (OR 0.58, 95 % CI 0.42–0.81, *P* = 0.001).

**Table 4:** Univariate and multivariate logistic regression show factors that predict dissatisfaction of eye care

	Univariate			Multivariate		
Variables	OR	95 % C.I.	*P* value	OR	95 % C.I.	*P* value
Sex (male)	1.14	(0.86–1.50)	0.353	–	–	–
Age (50–60 yrs)	0.78	(0.59–1.03)	0.075	0.75	(0.54–1.06)	0.107
Village (neighborhood of healthcare facility)	4.00	(2.97–5.38)	< 0.001	3.88	(2.85–5.28)	< 0.001
Years of residence (< 40)	0.74	(0.54–1.01)	0.059	0.77	(0.53–1.11)	0.163
Education (≤ junior high school)	1.11	(0.81–1.47)	0.473	–	–	–
Marital status (married/cohabiting)	0.86	(0.61–1.21)	0.382	–	–	–
Occupation (working group)	1.20	(0.91–1.59)	0.192	–	–	–
Religion (non-Buddist or Taoist)	0.64	(0.40–1.01)	0.054	0.70	(0.42–1.17)	0.174
Annual household income (≤ NTD 500,000)	1.30	(0.99–1.72)	0.063	1.27	(0.93–1.74)	0.131
Commercial insurance (yes)	0.92	(0.69–1.21)	0.542	–	–	–
Comorbid condition (no)	–	–	0.674	–	–	–
1 disease	1.03	(0.74–1.42)	0.870	–	–	–
2 diseases	1.01	(0.67–1.51)	0.980	–	–	–
at least 3 diseases	0.74	(0.44–1.26)	0.268	–	–	–
Alcohol (no)	1.09	(0.82–1.44)	0.556	–	–	–
Tobacco (no)	1.26	(0.88–1.82)	0.207	–	–	–
Daily sun exposure time (< 3 hours)	0.66	(0.48–0.92)	0.015	0.81	(0.56–1.15)	0.235
Composite score of NEI-VFQ-25 (≤ 85)	0.60	(0.45–0.80)	0.001	0.58	(0.42–0.81)	0.001

Items in parentheses indicate reference group

*Abbreviations*: *NTD* New Taiwan dollar, *NEI-VFQ-25* 25-item National Eye Institute Visual Function Questionnaire

The reliability for NEI-VFQ-25 was 0.94 (Cronbach’s α value). The relationships between common eye diseases and the composite and subscale scores of NEI-VFQ-25 are summarized in Table 5. The higher the VRQoL, the less inconvenience and fewer difficulties were reported by the participants in the questionnaire. As shown in Table 5, for all participants, the most inconvenience experienced was “general health” (42.8 ± 25.6), followed by “general vision” (62.7 ± 16.7), “vision specific role difficulties” (74.1 ± 23.6) and “ocular pain” (80.1 ± 17.8). The least inconvenience experienced was found for “color vision” (95.3 ± 14.1), followed by “driving” (95.0 ± 10.2), “vision specific social functioning” (93.8 ± 9.1) and “peripheral vision” (89.8 ± 14.2). For patients with cataracts, the composite score and all subscale scores were significantly lower than for patients without cataracts (*P* < 0.05). For patients with glaucoma, the composite and all subscale scores were significantly lower (*P* < 0.05) than for patients without glaucoma, except for the subscales of “general health” (*P* = 0.083), “near activities” (*P* = 0.124), “social functioning” (*P* = 0.185) and “color vision” (*P* = 0.346). For patients with dry eyes, the composite and all subscale scores were not significantly lower (*P* > 0.05) than for patients without dry eyes except for the subscale score of “ocular pain” (*P* < 0.001). Furthermore, we conducted multivariate linear regression on the composite score of NEI-VFQ-25 regarding predisposing and enabling factors and other predictors (Table 6). Predisposing factors (age, marital status, occupation), comorbid condition, enabling factors (residential village, commercial insurance, annual household income), cataracts and glaucoma were significantly associated with the composite NEI-VFQ-25 score. Older age (*P* = 0.021), not living in the neighborhood of the healthcare facility (*P* = 0.002), not being married or cohabiting (*P* = 0.035), being in the non-working group (*P* < 0.001), no commercial insurance (*P* = 0.009), higher annual household income (*P* = 0.012), more comorbid conditions (*P* < 0.001), cataracts (*P* < 0.001) and glaucoma (*P* = 0.046) were associated with lower composite NEI-VFQ-25 scores compared to their counterparts.

**Table 5:** Change of subscale and composite scores of NEI-VFQ-25 regarding various eye diseases

	All participants	Cataract			Glaucoma			Dry eye		
		Yes	No		Yes	No		Yes	No	
Subscale	Mean ± SD	Mean	Mean	*P* value*	Mean	Mean	*P* value*	Mean	Mean	*P* value*
General health	42.8 ± 25.6	35.4	48.7	< 0.001	37.7	43.3	0.083	39.9	43.3	0.181
General vision	62.7 ± 16.7	57.0	67.1	< 0.001	57.5	63.1	0.010	61.1	62.9	0.283
Ocular pain	80.1 ± 17.8	74.3	84.7	< 0.001	69.2	81.1	< 0.001	73.4	81.2	< 0.001
Near activities	84.3 ± 15.4	78.7	88.8	< 0.001	80.9	84.7	0.124	83.2	84.5	0.472
Distance activities	87.7 ± 12.2	81.2	92.9	< 0.001	80.6	88.4	0.001	86.9	87.9	0.574
Vision specific										
Social functioning	93.8 ± 9.1	90.3	96.6	< 0.001	91.6	94.0	0.185	94.5	93.7	0.534
Mental health	81.7 ± 14.6	76.6	85.8	< 0.001	75.9	82.3	0.006	80.9	81.9	0.569
Role difficulties	74.1 ± 23.6	66.4	80.2	< 0.001	61.6	75.3	< 0.001	70.2	74.8	0.088
Dependency	87.6 ± 14.8	81.4	92.6	< 0.001	81.9	88.2	0.026	86.6	87.8	0.573
Driving	95.0 ± 10.2	91.8	96.4	< 0.001	90.9	95.4	0.013	93.1	95.3	0.100
Color vision	95.3 ± 14.1	92.3	97.8	< 0.001	93.8	95.5	0.346	96.5	95.1	0.309
Peripheral vision	89.8 ± 14.2	84.4	94.1	< 0.001	85.3	90.2	0.031	89.1	89.9	0.632
Composite score of NEI-VFQ-25	84.0 ± 15.2	78.8	88.2	< 0.001	77.8	84.6	0.036	82.3	84.4	0.863

*Abbreviations*: *NEI-VFQ-25* 25-item National Eye Institute Visual Function Questionnaire, *SD* standard deviation

*P values indicate significance of Student’s t test

**Table 6:** Multivariate linear regression models of the relationship between vision-related quality of life (composite score of NEI-VFQ-25) and predictors

	Full model			Reduced model		
Variables	Coefficient	95 % C.I.	*P* value	Coefficient	95 % C.I.	*P* value
Intercept	112.38	98.51 ~ 126.25	< 0.001	120.77	113.97 ~ 127.56	< 0.001
Sex (male)	−0.68	−2.95 ~ 1.60	0.560	–	–	–
Age (50–60 yrs)	−3.01	−5.59 ~ −0.42	0.023	−2.81	−5.19 ~ −0.43	0.021
Village (neighborhood of healthcare facility)	−3.17	−5.05 ~ −1.29	0.001	−2.99	−4.85 ~ −1.13	0.002
Years of residence (< 40)	1.22	−1.09 ~ 3.53	0.300	–	–	–
Education (≤ junior high school)	0.42	−2.15 ~ 2.98	0.748	–	–	–
Religion (non-Buddist or Taoist)	2.83	−0.42 ~ 6.07	0.088	–	–	–
Marital status (married or cohabiting)	−2.45	−4.88 ~ −0.02	0.048	−2.56	−4.94 ~ −0.18	0.035
Occupation (working group)	−5.24	−7.57 ~ −2.90	< 0.001	−5.81	−8.02 ~ −3.59	< 0.001
Annual household income (≤ NTD 500,000)	−2.46	−4.50 ~ −0.43	0.017	−2.50	−4.44 ~ −0.56	0.012
Commercial insurance (yes)	−2.84	−5.03 ~ −0.65	0.011	−2.82	−4.94 ~ −0.70	0.009
Comorbid condition (< 2 diseases)	−4.66	−6.96 ~ −2.35	< 0.001	−4.78	−7.04 ~ −2.51	< 0.001
Alcohol (no)	0.51	−1.60 ~ 2.62	0.634	–	–	–
Tobacco (no)	–1.36	−4.16 ~ 1.45	0.343	–	–	–
Daily sun exposure time (< 3 hours)	1.67	–0.58 ~ 3.92	0.146	–	–	–
Cataract (no)	−4.90	−6.97 ~ −2.82	< 0.001	−5.04	−7.08 ~ −3.01	< 0.001
Glaucoma (no)	−3.24	−6.52 ~ 0.03	0.052	−3.32	−6.58 ~ −0.07	0.046

Items in parentheses indicate reference group

*Abbreviations*: *NTD* New Taiwan dollar

## Discussion

The most important contribution of this study is to explore the ophthalmic needs, accessibility and VRQoL provided by IDS on an alternating week shift basis for the residents of an outlying island. The results of this study indicated that 61.0 % of participants utilized ophthalmic care in the past year (indicator of realized access), and 17.6 % of participants reported unmet ophthalmic needs in the past 6 months. Satisfaction (another indicator of realized access) was estimated to be 88.1 %, suggesting that ophthalmic care under this system provided acceptable health service access that could somewhat bridge the gaps of unequal physician resources. Participants with cataracts or glaucoma perceived significant inconvenience and difficulties as described by the NEI-VFQ-25 compared to their counterparts. To the best of our knowledge, there is no literature assessing VRQoL and its association with common eye diseases on a community basis under IDS.

### Access of ophthalmic care under IDS

Currently, IDS offers a tentative solution to cope with the dearth of specialists or facilities in the mountainous areas and outlying islands of Taiwan, where residents often face shortages of services and inconvenient transportation. Although these residents of these remote areas (approximately 1.64 % of Taiwan’s population) may have nearly equal financial access, they may not have equal medical access because of the maldistribution or paucity of healthcare resources [15]. The IDS helped the people of the outlying islands of Taiwan to obtain the most appropriate ophthalmic care possible. In a systemic review of the current literature assessing the association between IDS and quality, the majority of studies have shown that IDSs have positive effects on the quality of care [1]. To achieve greater acceptance and satisfaction, providers and payers should consider patients’ needs and expectations while implementing innovative IDSs [26]. In addition to implementing IDS programs in these remote areas, healthcare authorities and the local government also need to encourage locals who have completed specialist training at medical centers to return to their hometowns to serve their neighbors. After all, healthcare services provided by physicians living locally are generally more readily available than services provided by physicians on shifts.

Notably, our findings might enrich the literature by suggesting factors influencing dissatisfaction with ophthalmic care under IDS (i.e., longer distances to the healthcare facility and lower VRQoL were associated with higher dissatisfaction). Tan et al. investigated accessibility of the Health Care Improvement Program in a mountainous district in rural Taiwan, and revealed patients who were not satisfied with the services of 24-h clinic were mainly from the most remote areas of the district [11]. Our result also supported a previous study that assessed patient satisfaction with perinatal healthcare [27]. That study found that the inconvenience of service utilization, such as distance to the medical facility, was inversely associated with satisfaction. However, another study regarding orthopedic outpatient satisfaction showed that travel distance was positively associated with patient satisfaction, with patients who lived closer reporting less satisfaction than patients who lived farther away, likely due to differences in the demographic composition (such as age) of the two groups [28]. The finding of our study that self-perceived VRQoL affected satisfaction with IDS was similar to a study that a positive health perception was significant in predicting satisfaction with healthcare service [29]. A recent study also demonstrated a strong association between self-perceived health and satisfaction with healthcare services [30].

The utilization of eye care under IDS in our study might be influenced by several factors, such as age, residential village, duration of residence, marital status and religion. Schaumberg et al. reported that the demographic predictors of eye care utilization within a 2-year period among women included age, education level, income, race/ethnicity, and region of residence [31]. Hong et al. reported that the utilization of eye care was 28.1 % among individuals with financial difficulty versus 41.9 % among individuals without [32]. Using a world-wide, population-based dataset, Vela et al. found that the rates of having had an eye exam in a year among older adults in low, lower middle, upper middle, and high income countries were 10, 24, 22, and 37 %, respectively [33]. Lack of eye care insurance for routine eye examinations might also have a negative impact on adolescents’ access to eye care providers [34]. We did find that a lack of commercial insurance was associated with more unmet ophthalmic needs. However, annual household income and commercial insurance did not influence eye care utilization in our study. The reason might be that the NHI program waived the copayment of the beneficiaries under IDS, thereby decreasing the financial inequity and increasing eye care utilization.

Cataract and glaucoma are the leading causes of blindness and visual impairment worldwide [35]. Rural residents are exposed to higher levels of ultra-violet radiation than their urban counterparts [3]. A recent study of population at age 40 years and older in Taiwan proper showed that the prevalence of cataracts as indicated by medical professionals (excluding mild cataracts) was 11.8 % [36]. The prevalence of moderate and severe cataracts in Matsu (13.9 %, 117/841) was consistent with this previous study, as we investigated residents aged 50 years and older. Glaucoma occurred in 8.7 % of the participants. This finding seemed higher than expected when compared to other neighboring countries, such as Japan (3.7 %, aged over 40 years) [37]. In southern China, glaucoma affected nearly 3 % of the population aged 50 years and over [38]. Our finding might be related to the efforts of an annual community-based integrated screening project including eye screening, which aims at the early detection of glaucoma and its suspects in Matsu. Laser treatment for glaucoma or surgery for cataracts is not currently available under IDS due to cost-benefit concerns. Patients with surgical ophthalmic conditions still need to be transferred to medical facilities in Northern Taiwan.

### VRQoL

The multidimensional nature of NEI-VFQ-25 allows it to measure multiple specific conditions in vision problems of varying severity [23, 24]. Our study showed similar results to the findings of Mangione in that NEI-VFQ-25 was sensitive to the visual disability of cataracts and glaucoma [24]. Glaucoma negatively affected the composite and several subscale scores of NEI-VFQ-25, and this effect was reported to be correlated with the severity of glaucomatous visual field loss [20]. Lin et al. employed NEI-VFQ-25 to evaluate the hospital-based VRQoL of glaucoma patients in a metropolitan area in northern Taiwan [23]. The highest mean score was “color vision”, followed by “social functioning”, “driving”, “peripheral vision”, “dependency”, and the lowest mean score was “general health”, followed by “general vision”, “role difficulties”. The rank of the subscales was similar to our findings of community-based participants with glaucoma in a rural area (Table 5). The situation might be because we were dealing with people with similar ethnicity, cultural behavior and lifestyles. The practice of the IDS eye care did scarcely alter the subscale rank of glaucoma on VRQoL despite rural-urban discrepancy.

The NEI-VFQ-25 could provide reliable, valid, responsive data on VRQoL for group-level comparisons [19]. In our study from a rural community perspective, participants with cataracts reported experiencing all subscales of inconvenience and difficulties related to visual function compared to their counterparts; likewise, participants with glaucoma reported experiencing over half the subscales of inconvenience and difficulties related to visual function compared to their counterparts (Table 5). Composite scores can be useful summaries of visual function [19]. The composite NEI-VFQ-25 score was significantly decreased in participants with cataracts or glaucoma compared to their respective counterparts. For decades, cataracts and glaucoma have been the major two causes that impair VRQoL in Matsu. This situation is consistent with the global trends of visual impairment when the population is under better healthcare [35]. The experience of using NEI-VFQ-25 to evaluate the VRQoL of common eye diseases in a community under IDS, as reported here, is successful, intuitive and informative.

### Limitations

We acknowledge several limitations in the current study. First, due to the design of the study, we did not retrieve the medical charts to validate eye diseases and comorbid conditions. However, the local administration checks residents’ health status annually (including eye diseases, especially focusing on cataract and glaucoma screening) and provides them with information and suggestions regarding the screening results. The participants should be well informed of eye diseases. Second, this census still omitted approximately 40 % of the residents of Matsu, as during the study period, many residents were employed in Taiwan or in Mainland China to make a living. Hence, the findings of this study are only suggestive. Third, although the satisfaction rate in our study was acceptably high, it indicates the popularity of the IDS program in ophthalmic care only. The generalizability of satisfaction with the IDS program to other specialty services warrants further evaluation.

## Conclusions

This study investigated the utilization of and satisfaction with ophthalmic care under the IDS and examined the factors influencing VRQoL for residents in the outlying island of Taiwan. To improve accessibility, Taipei City Hospital provides ophthalmic service under an IDS contract to satisfy the ophthalmic demand and remedy the specialist shortage of Matsu since 2000. Approximately 60 and 14 % of the participants reported their utilization of ophthalmic care and history of being transferred to Taiwan proper for further eye evaluation and care, respectively. The rate of satisfaction with the ophthalmic care provided by the IDS program is high, indicating that the current fortnightly 6-day ophthalmic service in Matsu is acceptable, predictable and sustainable. Residents who live in the neighborhood of the facility or who have high VRQoL are less likely to be dissatisfied with ophthalmic care under IDS. Future research may investigate whether IDS provides similar acceptance in terms of utilization and satisfaction in other specialty fields.

## Abbreviations

CI, confidence interval; IDS, integrated delivery system; NEI-VFQ-25, 25-item National Eye Institute Visual Function Questionnaire; NHI, National health insurance; NTD, New Taiwan dollar; OR, odds ratio; VRQoL, vision-related quality of life

## References

[1] Hwang W, Chang J, Laclair M, Paz H (2013). Effects of integrated delivery system on cost and quality. Am J Manag Care.

[2] Tsai WC, Kung PT, Yaung CL, Li YH, Lin SC (2006). Accessibility and satisfaction with healthcare by rural area residents. Taiwan J Public Health.

[3] Saliba AJ (2008). Impact of rurality on optical health: review of the literature and relevant Australian Bureau of Statistics data. Rural Remote Health.

[4] Schoenberg NE, Coward RT (1998). Residential differences in attitudes about barriers to using community-based services among older adults. J Rural Health.

[5] Andersen RM (1995). Revisiting the behavioral model and access to medical care: does it matter?. J Health Soc Behav.

[6] Andersen RM, McCutcheon A, Aday LA, Chiu GY, Bell R (1983). Exploring dimensions of access to medical care. Health Serv Res.

[7] Aday LA, Andersen RM (1981). Equity of access to medical care: a conceptual and empirical overview. Med Care.

[8] Chan WSH (2010). Taiwan’s healthcare report 2010. EPMA J.

[9] National Health Insurance Annual Report 2015-2016. http://www.nhi.gov.tw/Resource/webdata/13767_1_2015-2016%20NHI%20ANNUAL%20REPORT.pdf. Accessed 25 April 2016

[10] Hsiao CH, Ho CH, Liao CH, Wang HY, Wang JJ, Wu CC (2014). Wound dehiscence as a cataract surgery-associated postoperative complication in patients previously treated with alpha-1 blocker tamsulosin—a population-based study in Taiwan. Am J Ophthalmol.

[11] Tan HF, Tseng HF, Chang CK, Lin W, Hsiao SH (2005). Accessibility assessment of the Health Care Improvement Program in rural Taiwan. J Rural Health.

[12] Wen CP, Tsai SP, Chung WS (2008). A 10-year experience with universal health insurance in Taiwan: measuring changes in health and health disparity. Ann Intern Med.

[13] Keng SH, Sheu SJ (2013). The effect of national health insurance on mortality and the SES-health gradient: evidence from the elderly in Taiwan. Health Econ.

[14] Chen L, Yip W, Chang MC, Lin HS, Lee SD, Chiu YL (2007). The effects of Taiwan’s National Health Insurance on access and health status of the elderly. Health Econ.

[15] Lu JF, Hsiao WC (2003). Does universal health insurance make health care unaffordable? Lessons from Taiwan. Health Aff (Millwood).

[16] National Health Insurance in Taiwan 2011 Annual Report. http://www.nhi.gov.tw/resource/Webdata/20774_1_NHI%20IN%20TAIWAN%202011%20ANNUAL%20REPORT.pdf. Accessed 25 April 2016.

[17] Chen MK, Chen HH (2013). Increased cancer mortality in Taiwanese inter-island migrants. Geospat Health.

[18] Ahmad K, Zwi AB, Tarantola DJ, Chaudhry TA (2015). Self-perceived barriers to eye care in a hard-to-reach population: the Karachi Marine Fishing Communities Eye and General Health Survey. Invest Ophthalmol Vis Sci.

[19] Suzukamo Y, Oshika T, Yuzawa M, Tokuda Y, Tomidokoro A, Oki K (2005). Psychometric properties of the 25-item National Eye Institute Visual Function Questionnaire (NEI VFQ-25), Japanese version. Health Qual Life Outcomes.

[20] Nassiri N, Mehravaran S, Nouri-Mahdavi K, Coleman AL (2013). National Eye Institute Visual Function Questionnaire: usefulness in glaucoma. Optom Vis Sci.

[21] Orr P, Rentz AM, Margolis MK, Revicki DA, Dolan CM, Colman S (2011). Validation of the National Eye Institute Visual Function Questionnaire-25 (NEI VFQ-25) in age-related macular degeneration. Invest Ophthalmol Vis Sci.

[22] Lin JC, Chie WC (2009). Psychometric validation of the Taiwan Chinese version of the 25-item National Eye Institute Visual Functioning Questionnaire. J Eval Clin Pract.

[23] Lin JC, Yang MC (2009). Correlation of visual function with health-related quality of life in glaucoma patients. J Eval Clin Pract.

[24] Mangione CM, Lee PP, Gutierrez PR, Spritzer K, Berry S, Hays RD (2001). Development of the 25-item National Eye Institute Visual Function Questionnaire. Arch Ophthalmol.

[25] National Health Interview Survey 2005. http://nhis.nhri.org.tw/files/94NHIS_12-64.pdf. Accessed 25 April 2016.

[26] Juhnke C, Muhlbacher AC (2013). Patient-centredness in integrated healthcare delivery systems—needs, expectations and priorities for organised healthcare systems. Int J Integr Care.

[27] Senarath U, Fernando DN, Rodrigo I (2006). Factors determining client satisfaction with hospital-based perinatal care in Sri Lanka. Trop Med Int Health.

[28] Abtahi AM, Presson AP, Zhang C, Saltzman CL, Tyser AR (2015). Association between orthopaedic outpatient satisfaction and non-modifiable patient factors. J Bone Joint Surg Am.

[29] Jang Y, Kim G, Chiriboga DA (2005). Health, healthcare utilization, and satisfaction with service: barriers and facilitators for older Korean Americans. J Am Geriatr Soc.

[30] Paul P, Hakobyan M, Valtonen H (2016). The association between self-perceived health status and satisfaction with healthcare services: evidence from Armenia. BMC Health Serv Res.

[31] Schaumberg DA, Christen WG, Glynn RJ, Buring JE (2000). Demographic predictors of eye care utilization among women. Med Care.

[32] Hong CJ, Trope GE, Buys YM, Robinson BE, Jin YP (2014). Does government assistance improve utilization of eye care services by low-income individuals?. Can J Ophthalmol.

[33] Vela C, Samson E, Zunzunegui MV, Haddad S, Aubin MJ, Freeman EE (2012). Eye care utilization by older adults in low, middle, and high income countries. BMC Ophthalmol.

[34] Xu K, Trope GE, Buncic R, Jin YP (2012). Utilization of eye care providers by Canadian adolescents: evidence from the Canadian Community Health Survey. Can J Ophthalmol.

[35] Foster A, Resnikoff S (2005). The impact of Vision 2020 on global blindness. Eye (Lond).

[36] Shih YH, Chang HY, Lu MI, Hurng BS (2014). Time trend of prevalence of self-reported cataract and its association with prolonged sitting in Taiwan from 2001 and 2013. BMC Ophthalmol.

[37] Quigley HA, Broman AT (2006). The number of people with glaucoma worldwide in 2010 and 2020. Br J Ophthalmol.

[38] He M, Foster PJ, Ge J, Huang W, Zheng Y, Friedman DS (2006). Prevalence and clinical characteristics of glaucoma in adult Chinese: a population-based study in Liwan District, Guangzhou. Invest Ophthalmol Vis Sci.

